# Overview of Methods for Noise and Heat Reduction in MRI Gradient Coils

**DOI:** 10.3389/fphy.2022.907619

**Published:** 2022-07-08

**Authors:** Elizaveta Motovilova, Simone Angela Winkler

**Affiliations:** 1 Department of Radiology, Weill Cornell Medicine, New York, NY, United States; 2 Department of Radiology, Hospital for Special Surgery, New York, NY, United States

**Keywords:** MRI, gradient coil, vibroacoustics, acoustic noise, sound pressure level, MR safety, heating

## Abstract

Magnetic resonance imaging (MRI) gradient coils produce acoustic noise due to coil conductor vibrations caused by large Lorentz forces. Accurate sound pressure levels and modeling of heating are essential for the assessment of gradient coil safety. This work reviews the state-of-the-art numerical methods used in accurate gradient coil modeling and prediction of sound pressure levels (SPLs) and temperature rise. We review several approaches proposed for noise level reduction of high-performance gradient coils, with a maximum noise reduction of 20 decibels (dB) demonstrated. An efficient gradient cooling technique is also presented.

## 1 INTRODUCTION

Gradient coils of magnetic resonance imaging (MRI) scanners undergo large Lorentz forces as rapidly switched electrical currents are passing through them in the presence of the static magnetic field B_0_. Due to these forces, the gradient coil conductors vibrate, and these vibrations radiate into the air as acoustic pressure waves and sound radiation. The acoustic noise pattern depends on the gradient form and thus is different for each pulse sequence. The sound pressure levels (SPL) produced by gradient coils can exceed safety limits set by the National Institute of Occupational Safety (NIOSH) of 85 dB. For example, the echo-planar imaging (EPI) sequence, one of the loudest sequences, produces SPLs in the range of 110–120 dB. Exposure to such noise levels creates patient discomfort, anxiety, and could even result in a temporary hearing loss, thus requiring hearing protection for patients—and sometimes for operators [1]. High SPLs were also reported to cause other unwanted side effects, such as spectral line shape distortions, antisymmetrical sidebands, and signal loss [2, 3].

High-performance gradient coil designs that improve spatial/diffusion encoding and speed up data acquisition have been the focus of research in recent years [4–17]. However, these designs may further increase noise levels, as such systems are designed to produce stronger gradient fields and sharper slew rates. Moreover, the resulting gradient coil heating becomes an additional concern, as the surfaces of smaller insert coils are now much closer to the patients’ ears.

**FIGURE 1 |:**
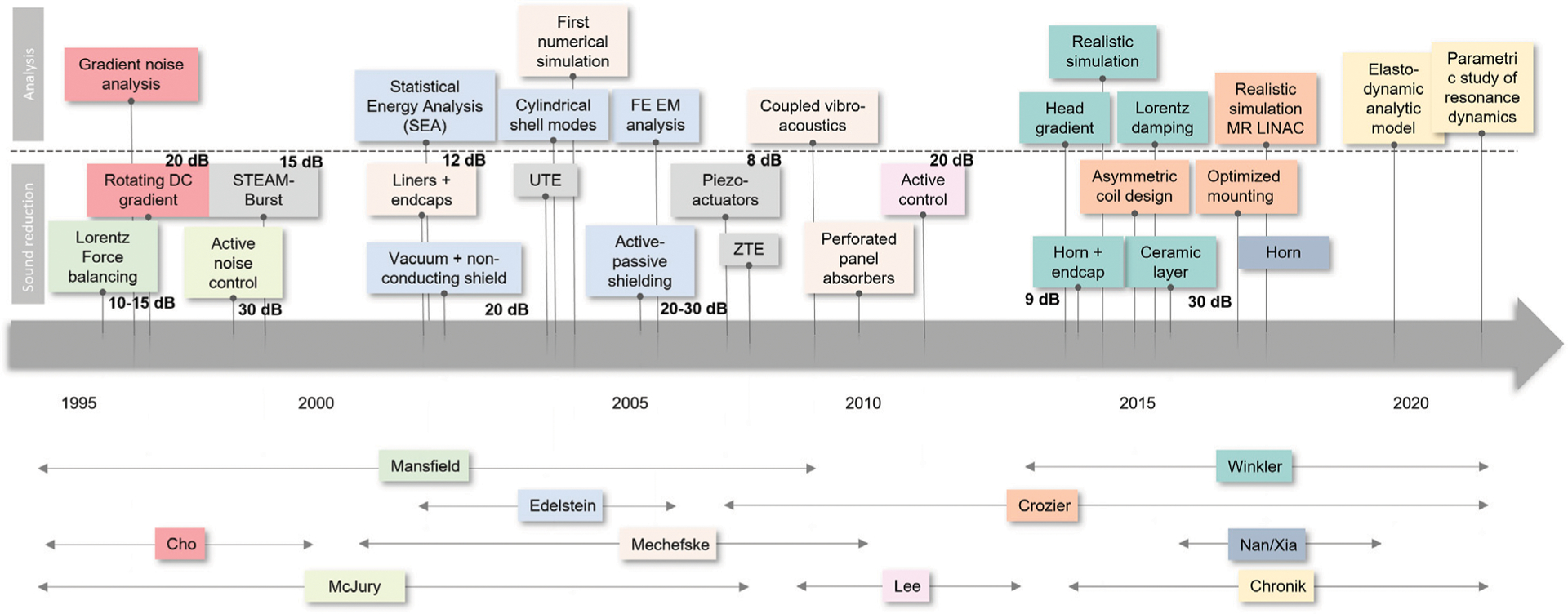
Timeline of analytical/numerical studies and noise reduction techniques for MRI gradient coils.

Therefore, accurate numerical modeling of gradient coil acoustics is essential for realistic noise estimates and safety analysis. Over the last two decades, many analytical, numerical, and experimental studies have been published to improve our understanding of vibrational properties of gradient coils. Several comprehensive review articles on the topic were published by McJury et al. in 2000 [18], Mechefske in 2008 [19], Takkar et al. in 2017 [20], Winkler et al. in 2018 [21], and most recently McJury provided a narrative/descriptive review in 2022 [22]. A summary of the analytical studies and noise reduction techniques is presented in Figure 1 in chronological order.

### 1.1 Analytical Models

The first analytical model to be used for gradient coil vibrations analysis was a thin-walled shell theory [23]. Taracila et al. calculated vibrational shell modes [24] and analyzed finite length cylindrical ducts with open end termination [25, 26]. In their comprehensive analysis [27, 28], Li and Mechefske combined both vibrational shell modes and acoustic wave propagation in open-ended cylindrical ducts. They were also the first to describe the coupling between the vibrational and acoustic modes.

### 1.2 Numerical Models

The first numerical model used to describe gradient coil acoustics was implemented using statistical energy analysis (SEA) by Edelstein et al. [1], which solves complex acoustic systems as an energy balance problem with a highly reduced computational cost compared to more detailed methods such as the finite element method (FEM). Mechefske et al. performed the first FEM numerical modeling of gradient coil acoustics, where both vibrational analysis and acoustics of a stand-alone thick-walled gradient cylinder were analyzed [27, 29–31]. FEM analysis was also used in a recent study for a split MRI-LINAC system [32, 33].

The computational resources of earlier studies were limited, and thus the first numerical models included assumptions and simplifications, such as neglecting certain physical effects. In [34] Winkler et al. proposed a new vibroacoustic model, which includes previously neglected but essential Lorentz damping along with additional previously neglected detail such as accurate wire patterns, the bore shape, patient bridge, and the air outside the bore. This realistic multiphysics simulation platform improves our understanding of the underlying principles of vibroacoustics in head gradient coils. Moreover, this simulation platform can help improve existing gradient coils and guide the design of novel gradient coils with lower SPLs.

Recently, Sakhr and Chronik proposed an exact linear analytical elastodynamic model for shielded longitudinal gradient coils [35]. This model demonstrates that the frequency response depends on a dimensionless “profile function” that specifies how the current density varies along the cylinder axis of the gradient coil. The model was then used to study the resonance dynamics of a gradient coil with respect to cylinder geometry parameters such as length, mean radius, and radial thickness [36].

### 1.3 Acoustic Noise Measurements

High acoustic noise levels in MRI have always been a source of safety concerns [18, 22, 37] and various noise reduction techniques have been proposed over the years. In 1995, McJury [37] measured the acoustic noise levels experienced during typical MRI sequences in 1.0 Tesla (T) and 1.5 T systems and found that many sequences produce noise levels above the regulatory safety thresholds. In 1997, Cho et al. [38] systematically studied the acoustic noise behavior of a commercial 1.5 T and a research-type 2.0 T systems using typical sequences such as Prescan, Spin-Echo (SE), Gradient-Echo (GE), Echo-Planar Imaging (EPI), and Inversion Recovery (IR). They found that the noise profile is not only dependent on the sequence parameters, but also on the gradient coil structure and support. They suggested two possible solutions to reduce the acoustic noise: 1) develop quieter imaging sequences, and/or 2) modify gradient coil structure. In [39], Mechefske measured the actual sound radiation experienced by patients at 4 T and proposed to use acoustic lining for noise reduction.

### 1.4 Silent Gradient Sequences

Among other methods, the acoustic noise in MRI can be reduced by optimizing pulse sequence parameters, e.g., the gradient slew-rate and amplitude. Cho et al. [40] developed an MRI technique based on projection reconstruction variation and using a mechanically rotating direct current (DC) gradient coil, which minimized gradient pulsing and resulted in a 20.7 dB noise attenuation. Another quiet gradient sequence called stimulated-echo acquisition mode (STEAM)-Burst was developed by Cremillieux et al. in 1997 and was 15 dB quieter than a typical EPI sequence [41]. Ultra-short time to echo (UTE) [42] and zero TE (ZTE) [43] sequences use radial sampling of k-space, and given the short repetition times (TRs) the gradients can remain active instead of requiring repeated switching, which results largely reduced acoustic noise in imaging procedures.

### 1.5 Active noise control

Several active noise cancellation (also called ‘antinoise’) techniques have been proposed over the years [44–49]. McJury et al. proposed an active noise control (ANC) system [45], in which the acoustic reduction of noise is achieved by introducing an antiphase acoustic wave to create a zone of destructive interference at a particular area in space. On average, 10–15 dB of noise was removed over the frequency range of 100–350 Hz, with a maximum noise reduction of 30 dB. Chen et al. [46] used a similar adaptive technique and achieved a noise reduction of 18.8 dB for frequencies below 4 kHz. Li et al. used an improved ANC system [47, 48] that works in a wide range of frequencies up to 5kHz, and allows the covering of most frequencies used in a typical MRI scanner. Chambers et al. [49] developed an opto-acoustical transducer that operates on the principal of light modulation and does not create electromagnetic interference (EMI), which is important for functional MRI.

### 1.6 Quiet Gradient Coils

Despite these methods that show varied success, the ideal approach to reduce acoustic noise in MRI is still the tackling of the source of the problem by designing “quiet” gradient coils. Gradient coils can be designed such that the Lorentz forces produced by the pulsing currents are balanced. Mansfield was the first to propose Lorentz force balancing [50–52] in 1994 with a 10 dB reduction achievable at 1.0 kHz. Active acoustic control [53, 54] operating at spot frequencies within a narrow band offered an average reduction in measured acoustic output of 30 dB.

Edelstein et al. used a combination of 1) a vacuum enclosure for gradient assembly isolation, 2) a radiofrequency (RF) coil with a low-eddy-current profile, and 3) an inner bore cryostat made of non-conducting material for acoustic noise reduction [1]. In the proposed active-passive shielding technique, it was also demonstrated numerically that the mechanical power deposition in the warm bore can be effectively decreased by wrapping a thin copper layer around the Z-gradient coil, which resulted in acoustic noise reduction of about 25 dB [55].

Roozen et al. [56] developed an active vibration noise control technique based on seismic mass piezo actuators, that reduced the spatially averaged acoustic noise of the Y-gradient coil vibrations by 3–8 dB at the dominant frequencies.

Wang et al. [57] proposed an asymmetric half-connected gradient coil design that improves the electromagnetic performance of the coil and provides higher efficiency, lower inductance, lower resistance, a higher figure of merit, and more acoustic radiation attenuation compared to the non-connected coils.

### 1.7 Passive Solutions

The mechanical vibrations of gradient coils can be attenuated by surrounding the coil with special noise absorbing materials for acoustic noise dampening.

Li and Mechefske showed that micro-perforated panel (MPP) acoustic absorbers, when properly designed, can reduce acoustic noise [58]. It was demonstrated experimentally that MPPs have multiple absorption frequency bands as well as wider frequency bands at higher frequency ranges [58].

Nan et al. proposed a technique for acoustic noise reduction in a split gradient coil [59], where 1) an asymmetric coil design was used to avoid vibrations of some resonant modes, and 2) horn structures were attached to the outer ends of the split main magnet such that they guided acoustic waves away from patient region.

While there are excellent review articles offering a comprehensive survey of the numerical methods for gradient coil analysis [21], as well as active acoustic control solutions such as ANC, “quiet” gradient coils, and “silent” pulse sequences [18, 22], other passive acoustic noise reduction methods are not well described in the literature. The purpose of this review is to summarize recent progress on acoustic noise reduction techniques with a focus on those using passive absorbing linings, and acoustic guiding structures. We also briefly discuss improved coil cooling strategies. The pursuit of quieter gradient coil technology remains a challenging area in MRI research and can potentially revolutionize medical imaging practice.

## 2 ACCURATE NUMERICAL MODELLING OF GRADIENT COILS

Accurate numerical modeling of gradient coils is essential for producing realistic predictions of SPLs. We numerically study and analyze gradient coils using a comprehensive numerical modeling approach where gradient-induced acoustics and vibrations are analyzed together with previously neglected but essential Lorentz damping. SPLs incurred by body and head gradient coils are compared. We also study how the strength of the main field B_0_ affects acoustic noise and vibration levels. We then focus on SPL reduction and efficient gradient cooling methods. To justify the numerical analysis, SPLs were also measured experimentally.

**FIGURE 2 |:**
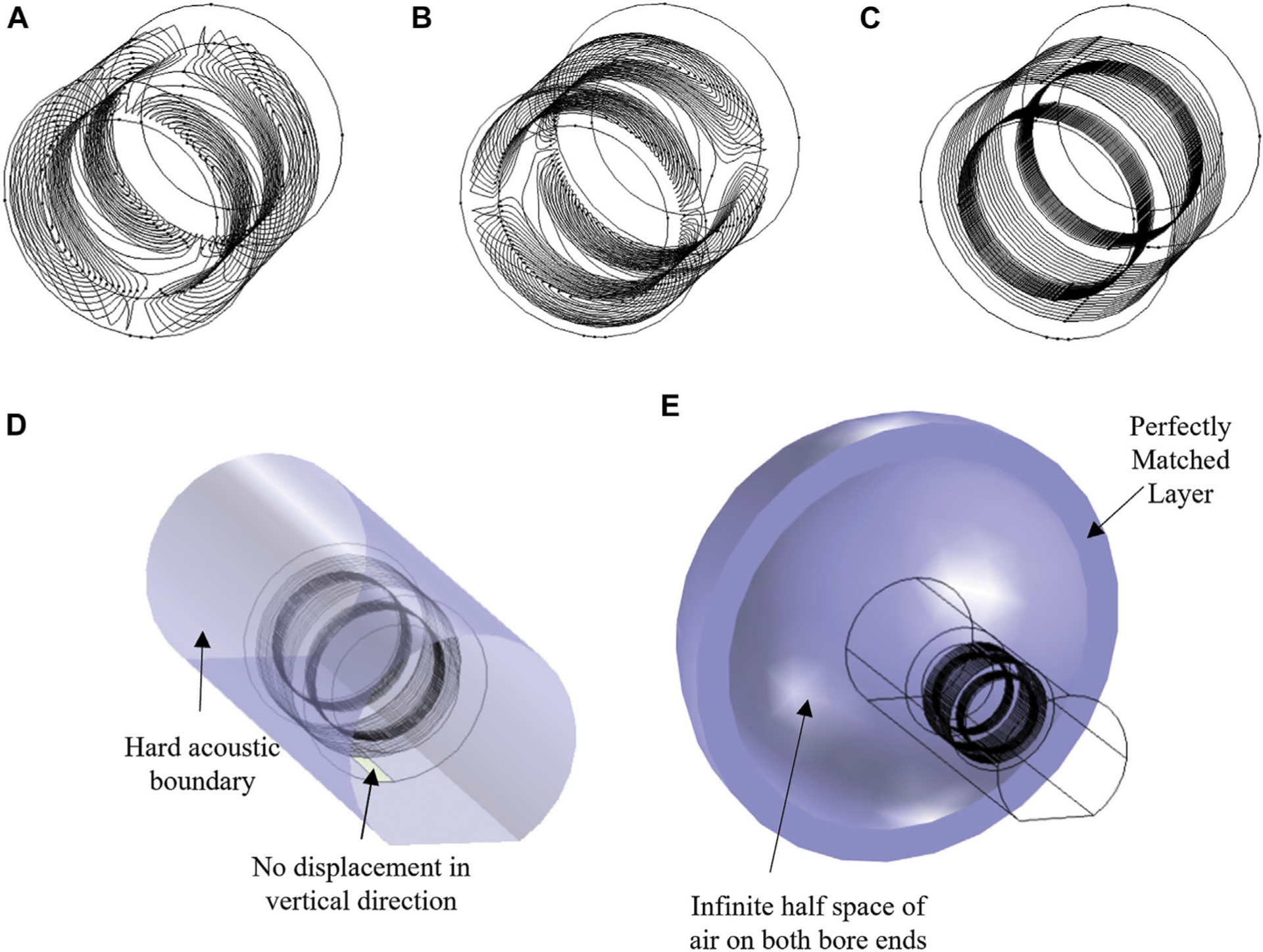
Simulation model. (**A**) X-, **(B)** Y-, and **(C)** Z-axis gradient coils. **(D,E)** Applied boundary conditions.

**FIGURE 3 |:**
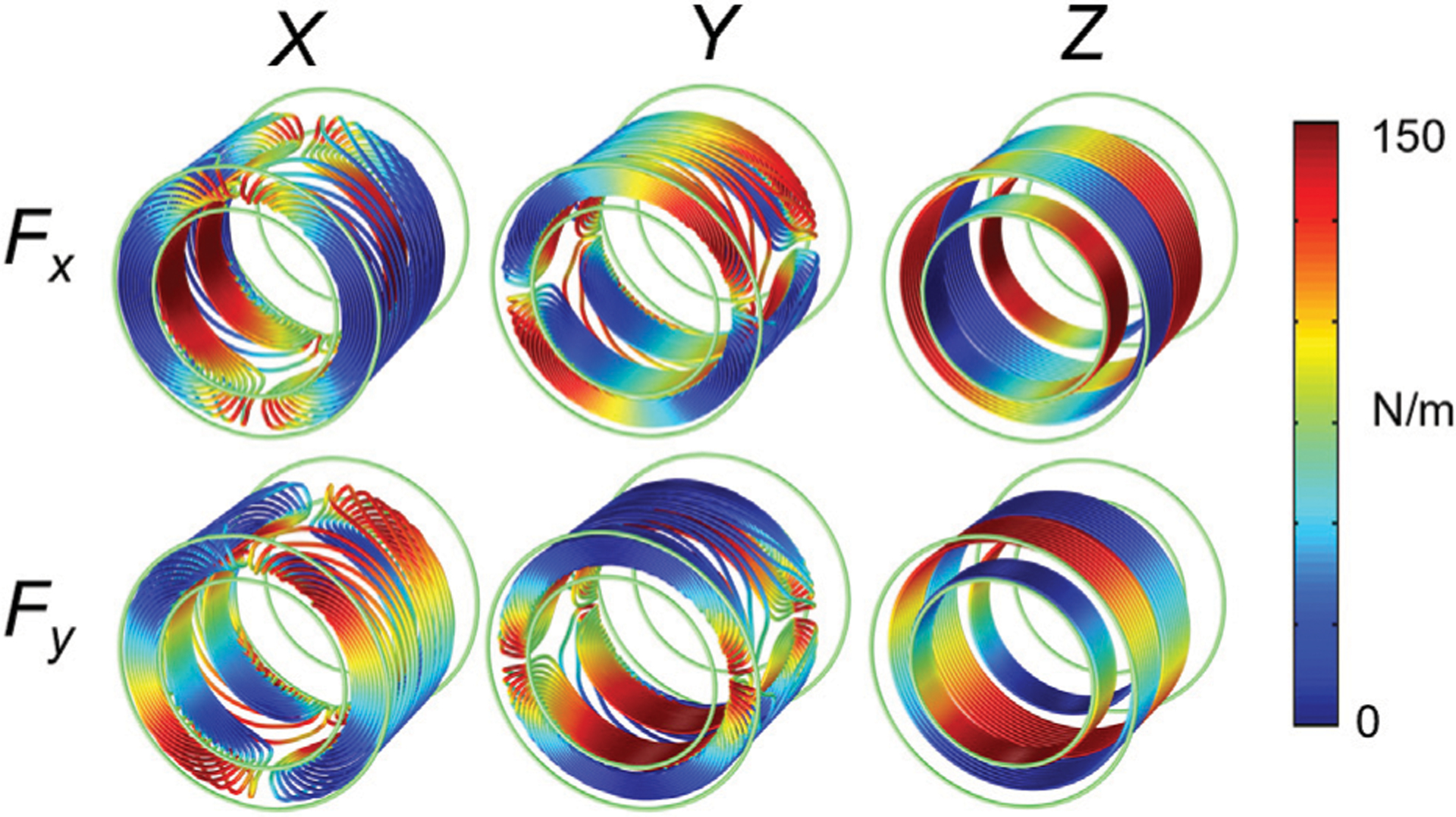
Lorentz forces on the gradient coil.

All numerical simulations were done using the finite-element package COMSOL. A folded shielded gradient head coil design intended for high performance human brain imaging was used as the base model [10]. The coil support structure was modeled as a cylinder with the following dimensions: inner diameter = 338 mm, outer diameter = 490 mm, length = 450 mm), and linear elastic material properties (*E* = 13 GPa, density *ρ* = 1,600 kg/m^3^, *ν* = 0.4). Conductor wire patterns were designed to accurately represent the spatial excitation distribution (Figures 2A–C). The air inside and outside the bore was modeled as a pressure acoustic fluid domain (speed of sound *c*_0_ = 343 m/s, *ρ* = 1.2 kg/m^3^). Full coupling between acoustics and structural vibrations was implemented in the simulation model. The MRI bore duct was modelled as a 60 cm diameter cylinder with a flat bottom (to represent the patient table support) with hard sound wall conditions (Figure 2D). At both ends of the bore, a hemispherical air volume of radius 1 m was added to simulate the sound waves propagation outside the bore. A perfectly matched layer (PML) of 20 cm was added to the model to mimic infinite size simulation domain (Figure 2E). A harmonic excitation with an alternating current (AC) of amplitude 50 A was used to drive the gradient coil. The frequency range of 0–3 kHz was chosen to cover the most pulse sequences used in MRI scanners. The performance of this head gradient coil was evaluated in comparison to the existing body gradient coil along the *X*-axis. Moreover, the head coil performance was evaluated and compared at three field strengths:−3, 7, and 10.5 T. Figure 3 shows the Lorentz forces **Fx** and **Fy** induced on all three gradient coils.

**FIGURE 4 |:**
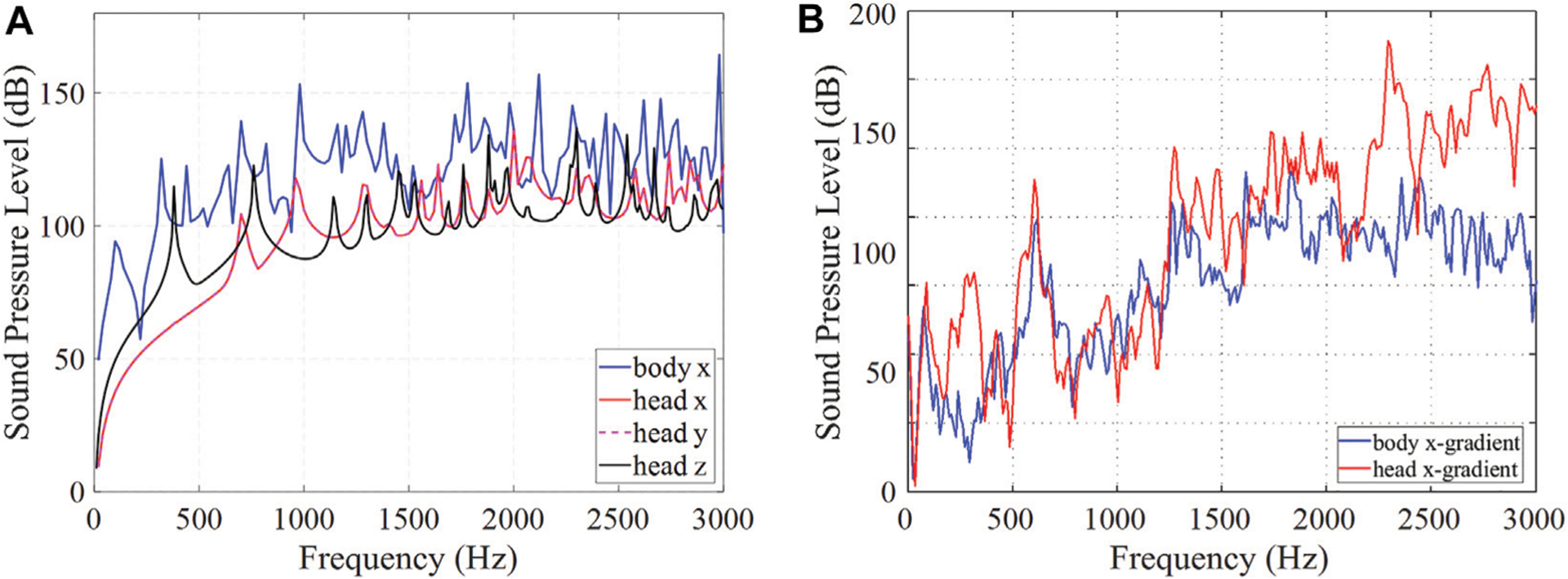
Comparison of head and body gradient coil spectra. **(A)** Standalone and **(B)** complete analysis.

Figure 4 compares SPL spectra of the head and body gradient coils performed using (a) a standalone analysis and (b) the complete realistic analysis. From the standalone analysis it appears that the body coil is louder as it has more excited modes in the spectra. However, the full analysis shows similar acoustic SPLs of the head and body gradients, with the average SPLs of 97.6 and 90.5 dB for the head and body gradient coils, respectively.

## 3 LORENTZ DAMPING AND MAGNETIC FIELD DEPENDENCE

One of the interesting predictions of the vibroacoustic model described in [34] is related to the dependence of the SPL on the main magnetic field *B*_0_. In [34], a motion equation for an incremental section of a conductor was derived with the secondary Lorentz force (a counter-EMF) taken into account. From there it follows that while the primary Lorentz force term depends linearly on the main magnetic field *B*_0_, the Lorentz damping term depends quadratically on *B*_0_. It indicates that the SPLs do not scale linearly with *B*_*0*_, as previously thought. In fact, if the damping term becomes large enough, the SPLs will decrease with *B*_*0*_.

**FIGURE 5 |:**
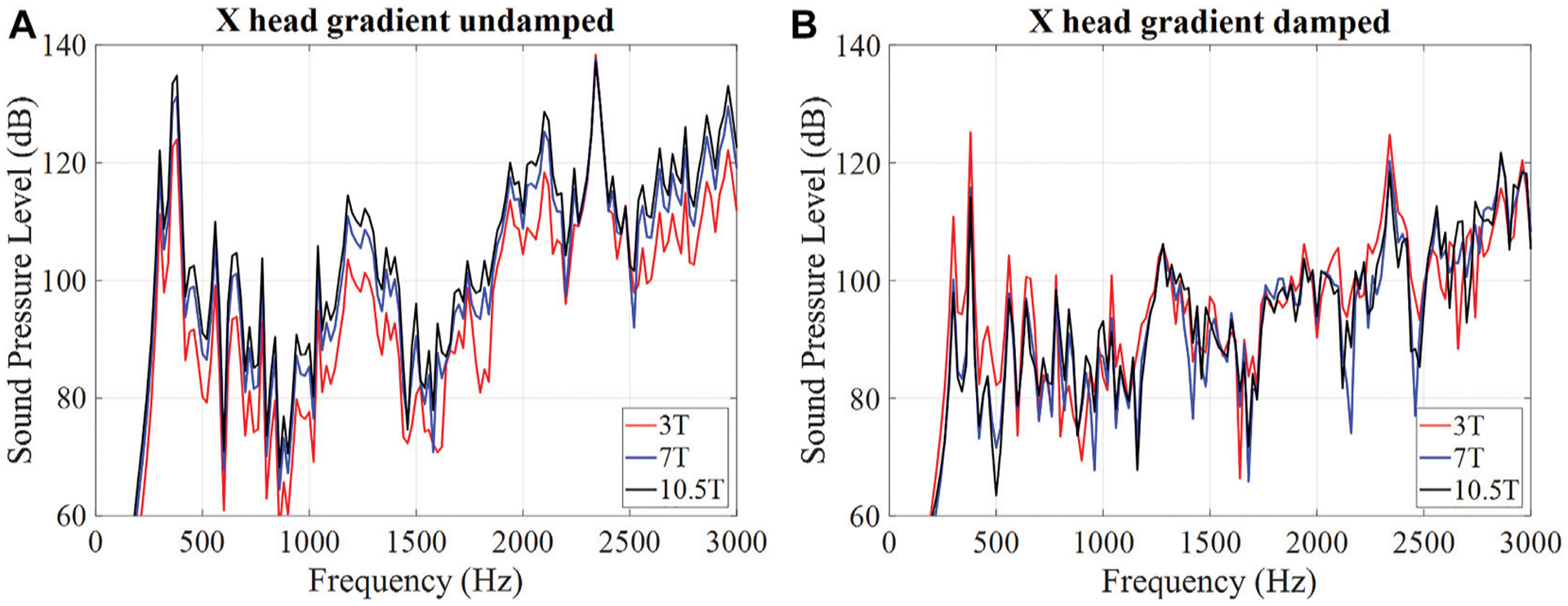
SPL spectra **(A)** without and **(B)** with Lorentz damping at different field strengths.

Figure 5 shows the simulated SPL spectra of the head gradient coil (X-axis only) (a) without and (b) with the added Lorentz damping term, respectively. If the Lorentz damping effect is not taken into account (a), the spectrally averaged SPLs were calculated to be 91.2, 97.5, and 100.8 dB, for 3, 7 and 10.5 T, respectively, confirming the expected linear scaling with the main field strength *B*_*0*._ However, some frequency points do not obey the linear relationship due to suspected structural-acoustic coupling. If the Lorentz damping is taken into account (b), the spectra behaves quite differently, with the calculated spectrally averaged SPLs of 92.1, 89.8, and 90.5 dB for 3, 7, and 10.5 T, respectively, demonstrating the reduction of the SPL values with the main magnetic field *B*_*0*_, as predicted by our improved numerical model.

## 4 NOISE REDUCTION METHODS

This section presents several methods for acoustic noise reduction in the MRI head gradient coil, including those using an absorbing foam and ceramic layer in various geometries, a horn sound guide, and endcap absorbers.

### 4.1 Absorbing Foam

**FIGURE 6 |:**
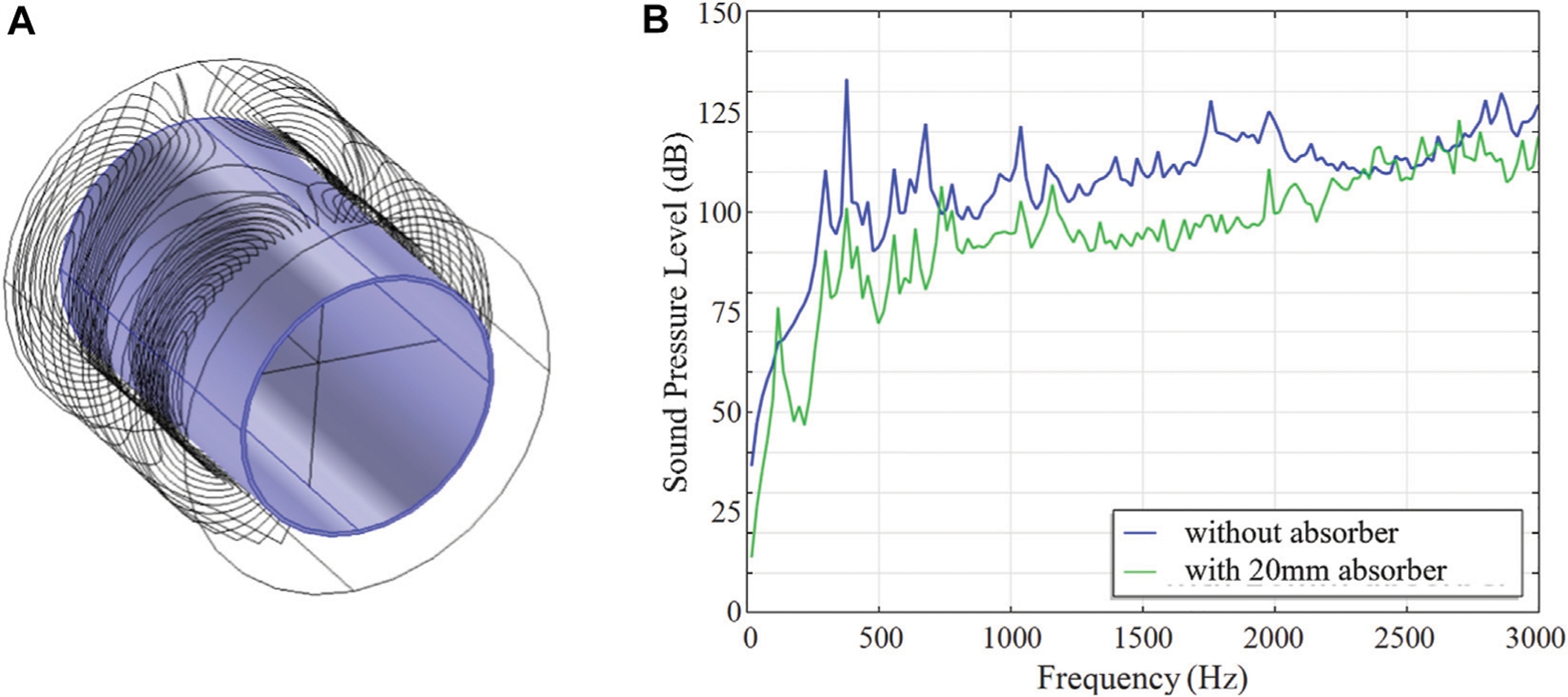
Noise reduction with an absorbing foam. **(A)** 3D model of the gradient coil and the absorber. **(B)** Simulated SPL results with and without the absorber.

Acoustic noise can be reduced with a layer of absorbing foam lining the inner bore wall as illustrated in Figure 6A; for example, with B-QUiet by VComp, foam layer thickness 20 mm, which provides a transmission loss of 15–37 dB in the range of frequencies from 125Hz to 4 kHz. Simulated bore volume average SPL spectra are shown in Figure 6B, with an average noise reduction of 12.5 dB (from 100.7 to 94.9 dB).

### 4.2 Ceramic Absorber

**FIGURE 7 |:**
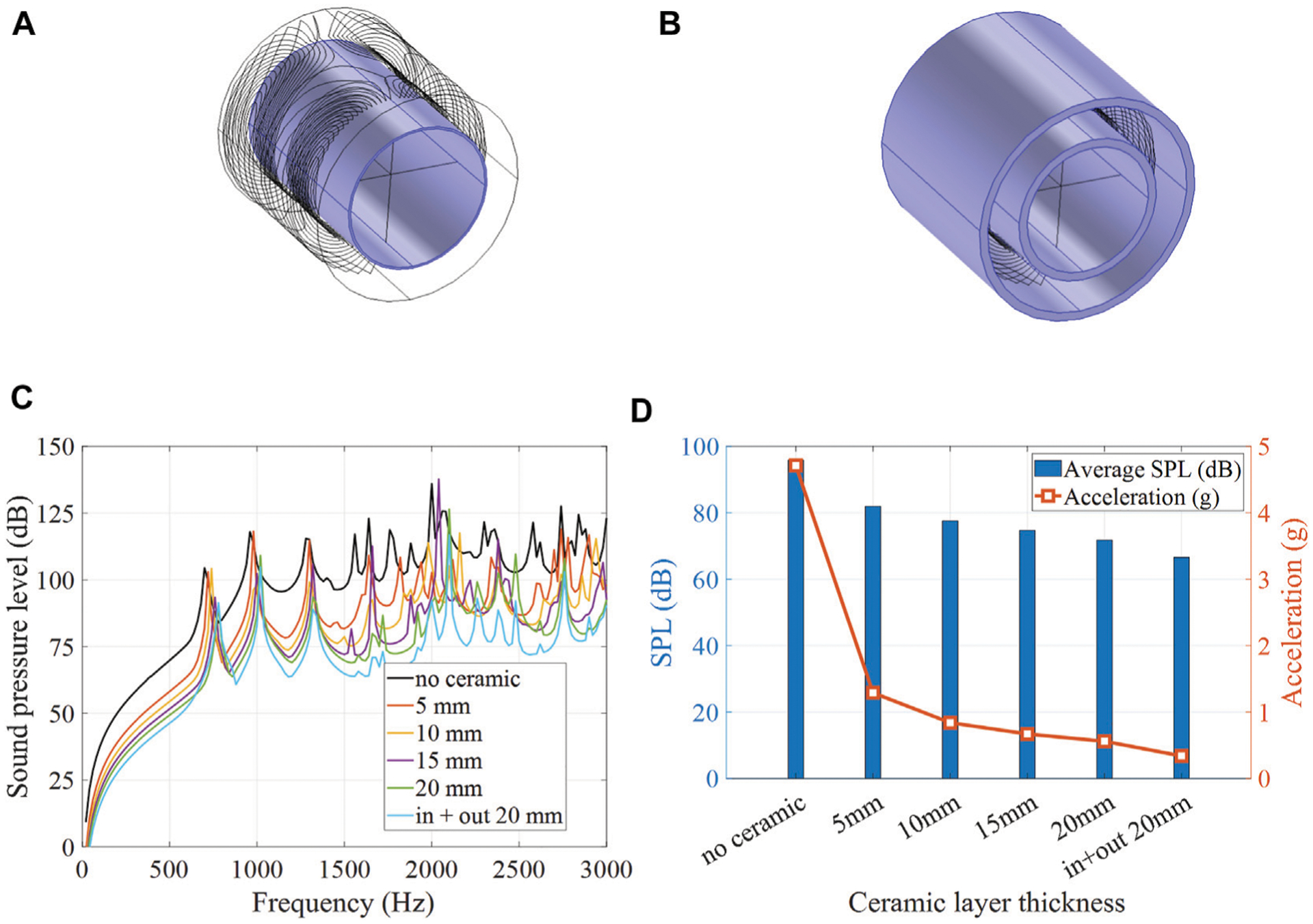
Noise reduction with an absorbing ceramic. 3D models of the gradient coil with **(A)** a single inner layer absorber and **(B)** inner and outer layer absorbers. **(C)** Simulated SPL spectra at various absorbing layer thicknesses. **(D)** Simulated average SPL and acceleration at various absorbing layer thicknesses.

Further, acoustic noise can be significantly reduced with a ceramic absorbing layer. A cylindrical ceramic layer (98% alumina, *E* = 300 GPa, density *ρ* = 3900 kg/m^3^, Poisson ratio ν = 0.22) of various thicknesses ranging from 5 to 20 mm was placed along the inner bore lining, as illustrated in Figure 7A. Moreover, a double layer configuration with two ceramic absorbers lining the inside and outside of the gradient coil was also considered, as illustrated in Figure 7B. Simulated bore volume averaged SPL spectra at various ceramic absorber thicknesses are shown in Figure 7C.Simulated averaged SPLs and acceleration at various ceramic absorbing layer thicknesses are shown in Figure 7D. It is demonstrated that frequency-averaged SPL reduction of 10.9 dB can be achieved with the help of a 20 mm ceramic layer insert alone, with the majority of noise reduction (10 dB) completed by the first 15 mm. With the addition of the outer ceramic layer, the average noise level is reduced by approximately 30 dB, from 95.8 to 66.6 dB.

### 4.3 Stepped Ceramic Absorber

**FIGURE 8 |:**
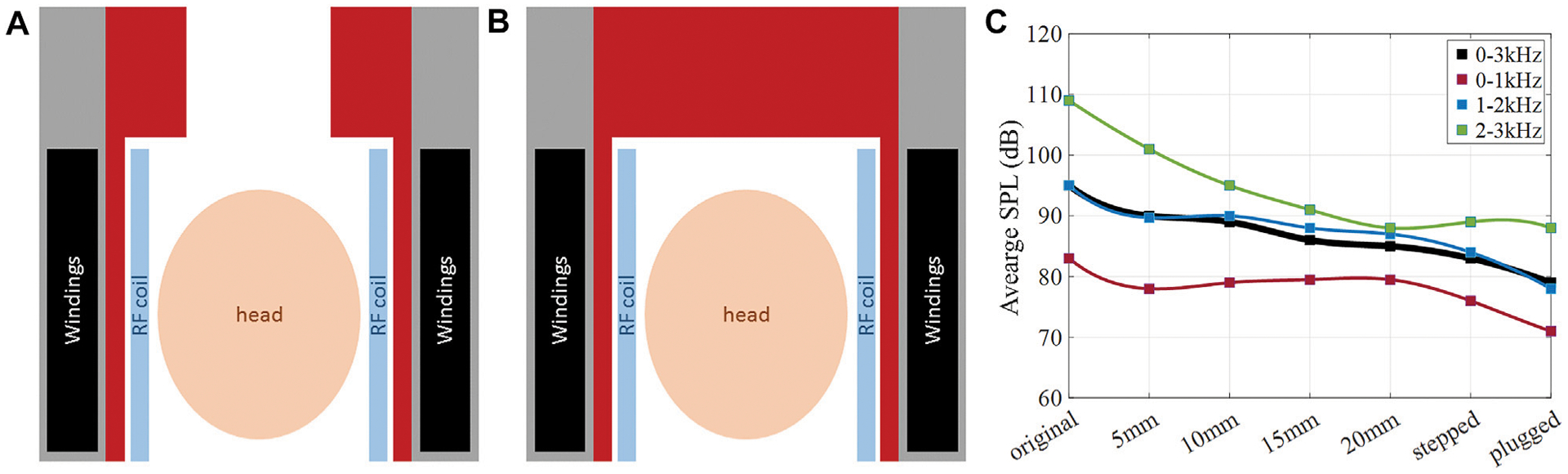
Noise reduction with ceramic layer of various shapes. 2D sketch of the head gradient coils setup with **(A)** a stepped ceramic insert, **(B)** a ceramic endcap. **(C)** Simulated average SPLs for the studied configurations.

Other ceramic layer configurations were considered as well. Figure 8 shows a 2D sketch of the head gradient coil with (a) a 20 mm ceramic absorber that includes an additional stepped section of 50 mm thickness extending over 200 mm at the service end of the gradient coil, and (b) a 20 mm thick cylinder in combination with a 200 mm thick “end-cap” completely filling the bore at the service end. Both designs (a) and (b) leave an adequate room to place the head and center the brain at the isocenter of the gradient coil. Figure 8C shows the frequency-averaged SPL reductions (black curve) due to the ceramic insert, with the maximum SPL reduction of 16.8 dB achieved with the plug insert. Moreover, frequency-averaged SPLs are shown for three separate frequency bands (red curve: 0–1 kHz, blue curve: 1–2 kHz, green curve: 2–3 kHz). The greatest SPL reductions are achieved in the high frequency band (green curve), with a maximum SPL reduction of 20.7 dB, the majority of which was reached by adding the first 15 mm of ceramic inner layer. This high frequency regime benefits the most from a ceramic layer alone without the need for stepped or plugged insert. In contrast, the lower frequency bands (blue and red curves) benefit the most from the added stepped or plugged ceramic insert, with SPL reduction in the range of 8 dB contributed by these features.

### 4.4 Horn and End Caps

**FIGURE 9 |:**
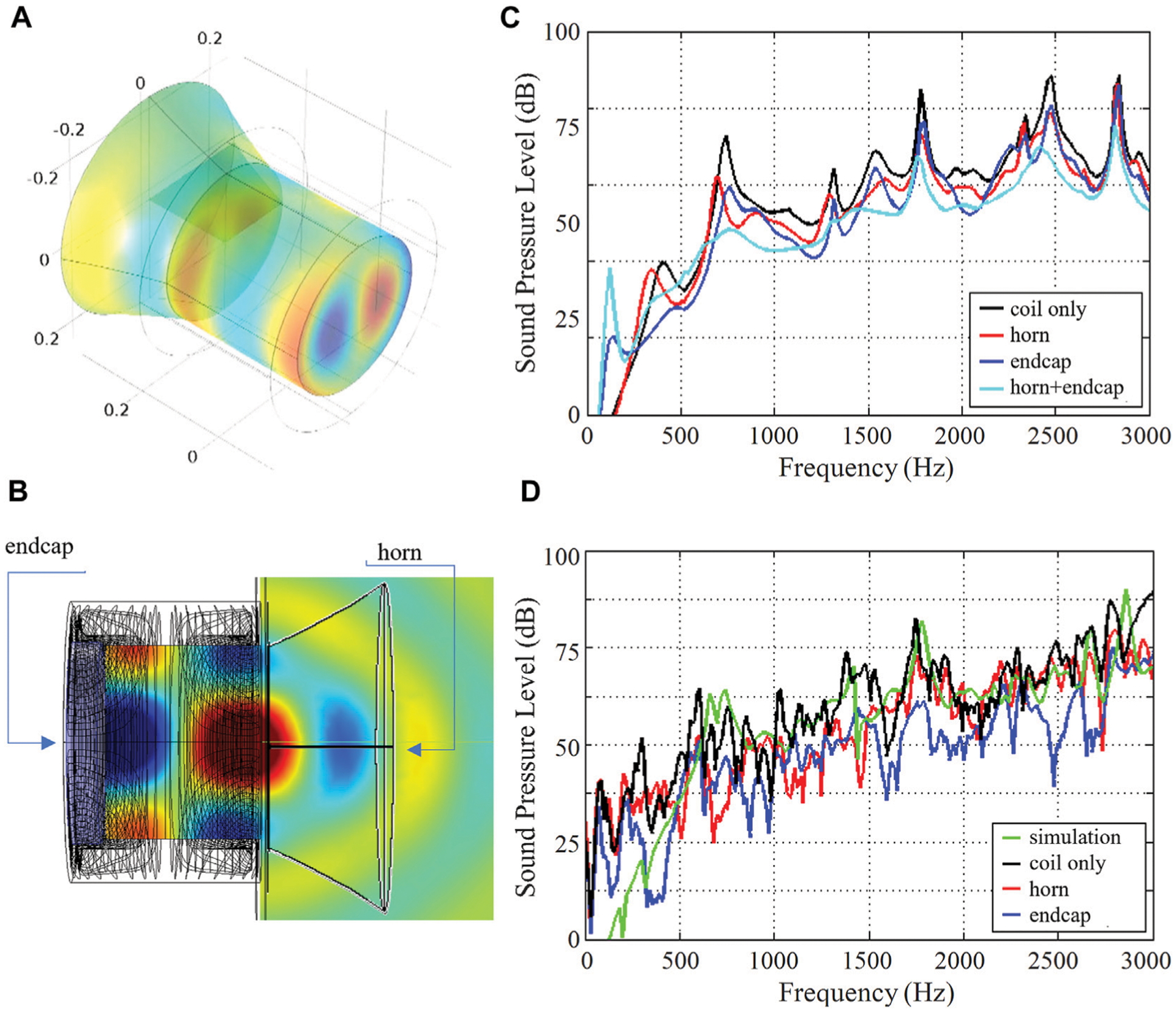
Noise reduction using a horn and endcaps. **(A)** 3D representation of a gradient coil with an additional horn structure for noise guidance and suppression. **(B)** 2D representation of the gradient coil with a horn and an end cap. **(C)** Simulated SPL spectrum. **(D)** Measured SPL spectra.

The MRI bore acts as an acoustic waveguide for sound waves. The bore ends introduce discontinuity in the sound wave propagation due to the change in the acoustic impedance at the interface of bore ends/outside air. In order to reduce acoustic SPLs inside the scanner bore, a horn structure could be used which flares out sound waves to better match the characteristic acoustic impedance of the MRI bore to the free space acoustic impedance of the outside air. The sound energy will then be better carried from the interior of the resonator toward the outside world, thereby reducing the acoustic energy resonating inside the bore. The horn model was studied in simulations using COMSOL. The horn shape was chosen to follow an exponential outline r (Z) = r_i_·*e*^*bz*^, which provides large impedance-matching bandwidth. Figure 9A illustrates this concept, where a horn is attached to one end of the bore and helps to guide the sound energy outward.

Another solution to minimize impedance discontinuity is to use an absorbing end cap at the bore end to absorb reflected energy. The absorbing end cap was studied in simulations using COMSOL. The end cap was modeled as a 5 cm thick cylinder with an absorption coefficient of 7 Np/m. Figure 9B illustrates this concept, where an end cap is attached to the other (service) end of the bore and absorbs the sound energy.

We used the same head gradient coil described in Section 2. The individual gradient axes were excited with a sinusoidal current waveform of 60 s sweep duration.

To confirm the simulation, experimental measurement of SPLs and vibration levels were performed. Sound pressure levels (SPLs) were measured using a Behringer ECM800 condenser microphone at various spatial positions in the bore. Vibration levels were measured using a single-axis Analog Devices ADX001–70Z accelerometer, sensitive to acceleration amplitudes of ±70 g. The accelerometer was positioned on the inner bore surface at various positions along the bore Z-axis.

Figure 9C shows the simulated SPLs averaged over the bore volume at each frequency point. The black curve corresponds to the unmodified gradient coil and shows an average of 77 dB over the band of interest (0–3,000 Hz). For the horn structure, it was found that the following parameters give the best impedance matching: *b* = 2, *h* = 10 cm. The simulated SPLs of the gradient coil with this horn structure are shown in red, with a calculated mean noise reduction of 2 dB. The simulated SPLs of the gradient coil with the end cap are shown in blue, with a calculated mean noise reduction of 2 dB. When the horn and end cap are used in combination (cyan curve), the mean noise reduction is 3 dB. Both techniques help to smooth out the peaks in the acoustic spectrum. The maximal noise reduction of 8, 10, and 13 dB was found at a frequency of 740 Hz for the horn, the end cap, and both of them used together, respectively. Figure 9D shows experimentally measured SPLs at a point +10 cm to the right of isocenter, for *X*-gradient excitation. The measured spectrum (black curve) agrees well with the simulation (green curve), with an average SPL of 76 dB. When the horn structure is used (red curve), the maximum recorded noise reduction is 28 dB with the average noise reduction of 4 dB across the whole frequency range. When the end cap is used (blue curve), the maximum recorded noise reduction is 27 dB, with the average noise reduction of 9 dB across the entire spectrum.

## 5 EFFICIENT GRADIENT COIL COOLING

High-performance MR gradient coils are subject to strong resistive heating due to the large electrical currents passing through them. For reasons of patient safety and system stability, it is important to limit the temperature rise inside the gradient coil and on the bore surfaces. In their recent work, Wade et al. [9, 10] demonstrated a novel insertable folded head gradient design for human brain imaging that uses hollow copper conductors to allow substantially higher thermal performance. To fully evaluate gradient coil safety, we require a tool for accurate thermal analysis, including the ability to model gradient axes built from either solid or hollow copper. Here we 1) model gradient configurations that use two hollow copper gradient axes and one solid copper axis in comparison with configurations that use all hollow copper axes, and 2) obtain spatially and temporally dependent temperature distributions to study and optimize the key design parameters of such gradient coils.

Realistic numerical modeling simulations were performed using COMSOL. The head gradient coil design described in Section 2 was used with the following modifications. Epoxy was modeled as a solid heat transfer domain (*ρ* = 1,600 kg/m^3^, *k* = 2.16 W/m.K, *C* = 1200J/kg.K). Accurate 3D hollow conductor paths were embedded in epoxy, with resistive heating modeled as a tubular heat source using the calculated copper resistive power dissipated per unit length in W/m, and with hollow conductor coolant flow modeled as a Newtonian fluid with inlet water temperature 16°C and flow rates of 1.74 L/min (*Y*) and 1 L/min (*Z*). We used conductor dimensions representative of one of the recently proposed head gradient prototypes (circular *Y*-conductor with inner diameter/wall thickness 3.5 mm/0.75 mm, rectangular *Z*-conductor with inner dimensions *w* = 2 mm and *h* = 4.5 mm and wall thickness of 0.75 mm) [9, 10]. Thermal performance was analyzed for two different scenarios: (a) full three-axis cooling with a flow rate on the *X*-axis of 0.72 L/min and a hollow copper conductor of inner diameter/wall thickness of 2.3 mm/1 mm, as well as (b) two-axis cooling with a solid *X*-axis conductor of varying diameters from 2 to 8 mm. All simulations were carried out using a DC excitation of 150 A on all three axes simultaneously over a transient period from 0 to 30 min.

**FIGURE 10 |:**
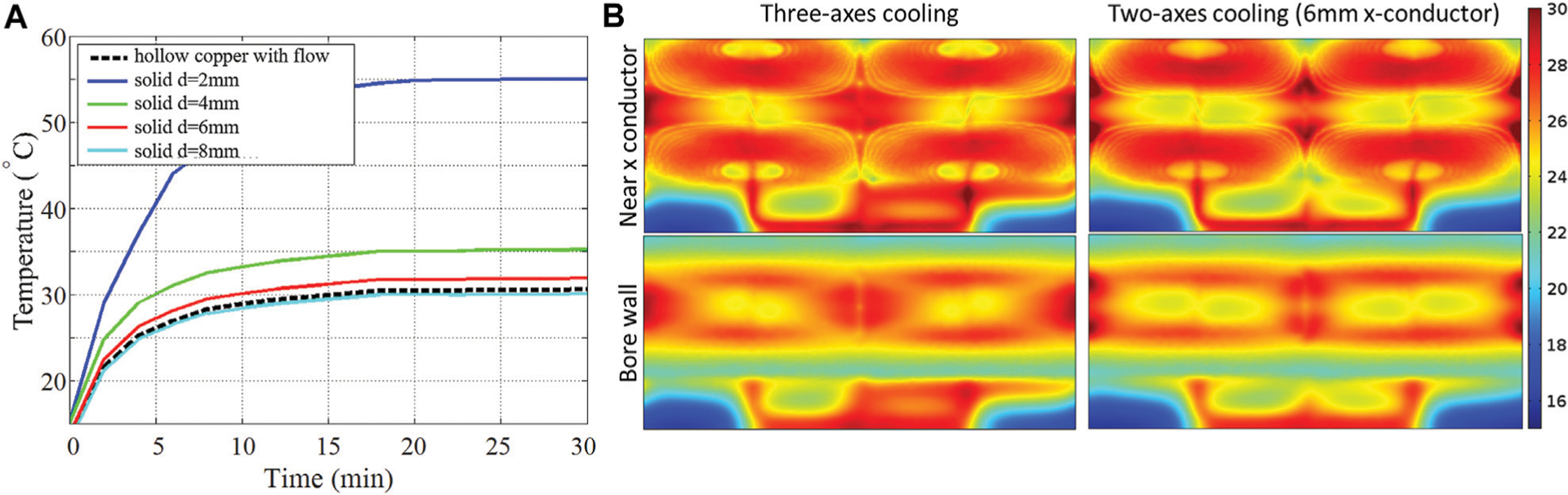
Efficient gradient cooling. **(A)** Temperature as a function of time. **(B)** Temperature distribution on the inner bore.

Figure 10A shows temperature as a function of time (*t* = 0–30 min) at a position embedded in the epoxy, close to the *X*-gradient conductors, at the top side, patient end of the head gradient (*x* = 0 mm, *y* = 189 mm, *z* = 157 mm). This position was estimated in advance to be among the hottest spatial locations. The black dashed line corresponds to full three-axis cooling and shows that a steady-state temperature of 30.5°C is reached. The colored lines correspond to two-axis cooling with solid *X*-conductors of diameters 2, 4, 6, and 8 mm, showing maximum steady-state temperature rise of 55.0, 35.1, 31.9, and 30.3°C, respectively. This result suggests that a two-axis (*Y, Z*) hollow conductor configuration with solid *X*-conductor of ≥6 mm diameter performs nearly as well as an all-hollow conductor coil of otherwise similar design. To further evaluate this equivalency, we show spatial temperature maps for the two configurations in Figure 10B; these maps show temperature distributions on cylindrical surfaces located at the inner bore wall (*r* = 165 mm) and near the *X*-conductor (*r* = 182 mm). The two maps show negligible differences, with r.m.s/maximum Δ*T* (temperature rise from the initial temperature of 15°C) of 9.5/15.8°C and 10.6/16.2°C for the three- and two-axis configurations, respectively.

## 6 DISCUSSION

In this paper, we show that numerical analysis of gradient coils can accurately predict vibroacoustics and temperature increase, which can further be used to reduce sound pressure levels and vibrations, ultimately leading to the safe operation of MR gradient coils. It was demonstrated that the Lorentz damping effect depends on the conductor cross section. At higher frequencies, when the skin depth is reduced, the amount of damping effect may reduce as well. Moreover, based on this realistic vibroacoustic modeling, the mechanical stress, vibrations, and SPLs of gradient coils might be much more manageable at ultra high fields (UHFs) than previously thought.

It was demonstrated that ceramic inserts provide significant SPL reduction in gradient coils. However, it should be noted that ceramic materials are relatively heavy, and the added weight has to be considered in any practical application. In particular, a 5 mm thick ceramic cylinder would add 20.4 kg to the gradient coil, while a 20 mm thick ceramic cylinder will add 35.1 kg. If the stepped ceramic absorber is used, the extra added weight goes up to 61.5 kg. The maximum added weight is for the plugged ceramic absorber design, with 89.5 kg extra weight added to the gradient coil. In terms of the most benefit per frequency, the additional plugged/stepped inserts show more noise reduction in the low-/intermediate-frequency bands, with the acoustic levels being 10–25 dB lower than in the high-frequency band. For the high frequency band, using a 15 mm straight ceramic layer will provide significant noise reduction, with an only moderately increase in weight. Moreover, the ceramic layer can improve thermal heat conduction and therefore minimize thermal hotspots.

It was shown that an end cap attached at the service end of gradient coil sound waves can absorb the acoustic noise while a horn structure attached to the other end can effectively guide the sound wave away from the bore into the free space. The combined effect of these two strategies applied together allows for a significant sound reduction.

We have presented a framework for the accurate thermal analysis of gradient coils. We demonstrate the feasibility of moving from an all-hollow copper (three cooled gradient axes) coil concept to a two-axis-only cooling concept that uses a 6 mm solid conductor for the third axis. The r.m.s/maximum temperature rise for the three- and two-axis configurations show negligible differences and are 9.5/15.8°C and 10.6/16.2°C, respectively.

Various acoustic noise reduction methods have been reported here and in the literature. From an engineering point of view, the best solution for tackling the acoustic noise problem is to redesign the gradient coil structure such that it does not produce unwanted noise; for example, by balancing out the Lorentz forces generated by the moving currents [50–52]. However, in practice, their installation into existing MRI systems could prove very expensive compared to other alternative noise mitigation strategies. Recent advances in “silent” MRI sequence developments such as UTE [42] and ZTE [43] are very encouraging with gradient noise levels of only 2.6 dBA above the in-bore background noise [60] and improved pediatric scan success rates [61]. However, some comparative reports indicate image blurring and reduced SNR for these sequences [62]. Modern ANC systems can achieve average SPL attenuation of 20 dB [63]. However, for optimal performance, such ANC systems have to be positioned very close to the patient’s ear, i.e., integrated into headphones. This creates a system limitation, as headphones are often incompatible with head coils. Passive acoustic noise reduction solutions, such as noise absorbing bore linings and end caps, can provide a considerable noise attenuation of up to 30 dB; however, their additional weight could become a design concern. The search for an elegant solution to gradient noise reduction is still active and ongoing, as a truly silent MRI system could potentially revolutionize medical imaging practice.

## 7 CONCLUSION

In this paper, we reviewed state-of-the-art numerical methods and practical solutions for acoustic noise reduction in MRI gradient coils. We provided a timeline outlining the major milestones in this research area, with the focus on passive noise reduction solutions using absorbing bore liners, endcaps, and other methods. We also discussed efficient cooling strategies. We highlight the importance of accurate and realistic multiphysics computational methods that include the previously neglected but essential Lorentz damping effect. Our analysis of the dependence of gradient vibroacoustics on the main magnetic field strength suggests that gradient acoustics and vibrations are more manageable at UHF MRI field strengths than previously thought. Experimental measurements of SPLs and acceleration levels agree well with the simulations. It was shown that a uniform 15 mm thick cylindrical ceramic insert is a practical design that provides a considerable acoustic noise reduction of 10.9 dB averaged over the frequency range of 0–3 kHz., with a substantially higher reduction of 20.7 dB in the high frequency range (2–3 kHz). Using horn and/or endcap results in only moderate noise reduction of 4 dB/9 dB averaged over 0–3 kHz.

## Data Availability

The raw data supporting the conclusions of this article will be made available by the authors, without undue reservation.
